# ﻿*Thyridium* revised: Synonymisation of *Phialemoniopsis* under *Thyridium* and establishment of a new order, Thyridiales

**DOI:** 10.3897/mycokeys.86.78989

**Published:** 2022-02-01

**Authors:** Ryosuke Sugita, Kazuaki Tanaka

**Affiliations:** 1 Faculty of Agriculture and Life Science, Hirosaki University, 3 Bunkyo-cho, Hirosaki, Aomori 036-8561, Japan Hirosaki University Hirosaki Japan; 2 The United Graduate School of Agricultural Sciences, Iwate University, 18-8 Ueda 3 chome, Morioka, Iwate 020-8550, Japan Iwate University Morioka Japan

**Keywords:** Ascomycota, Phialemoniopsidaceae, phylogeny, Sordariomycetes, taxonomy, Thyridiaceae

## Abstract

The genus *Thyridium*, previously known as a saprobic or hemibiotrophic ascomycete on various plants, was revised taxonomically and phylogenetically. Sequences of the following six regions, that is, the nuclear ribosomal internal transcribed spacer (ITS) region, the large subunit (LSU) of rDNA, the second largest RNA polymerase II subunit (*rpb2*) gene, translation elongation factor 1-alpha (*tef1*) gene, the actin (*act*) gene, and the beta-tubulin (*tub2*) gene, were generated for molecular phylogenetic analyses of species of this genus. *Phialemoniopsis*, a genus encompassing medically important species, is synonymised with *Thyridium* based on molecular evidence and morphological similarities in their asexual characters. The generic concept for *Thyridium* is expanded to include species possessing both coelomycetous and hyphomycetous complex asexual morphs. In addition to type species of *Thyridium*, *T.vestitum*, nine species were accepted in *Thyridium* upon morphological comparison and molecular phylogenetic analyses in this study. All seven species of *Phialemoniopsis* were treated as members of the genus *Thyridium* and new combinations were proposed. A bambusicolous fungus, *Pleosporapunctulata*, was transferred to *Thyridium*, and an epitype is designated for this species. A new species, *T.flavostromatum*, was described from *Phyllostachyspubescens*. The family Phialemoniopsidaceae, proposed as a familial placement for *Phialemoniopsis*, was regarded as a synonym of Thyridiaceae. A new order, Thyridiales, was established to accommodate Thyridiaceae; it forms a well-supported, monophyletic clade in Sordariomycetes.

## ﻿Introduction

*Thyridium* was originally established to accommodate species with cylindrical, uniseriate, 8-spored asci and muriform, dark-coloured, ascospores (Nitschke 1867). Species of this genus occur on various plants as saprobic or hemibiotrophic fungi (Eriksson and Yue 1989; Taylor et al. 1997; Checa et al. 2013). Currently, *Thyridium* includes 33 species and is placed in Thyridiaceae (family *incertae sedis*, Sordariomycetes; Yue and Eriksson 1987; Index Fungorum, http://www.indexfungorum.org, 2021). The type species *T.vestitum* has been verified to produce both coelomycetous and hyphomycetous asexual morphs, which have phialidic conidiogenous cells with collarette and ellipsoidal to allantoid hyaline conidia (Leuchtmann and Müller 1986).

Molecular information on *Thyridium* species is available only for two non-type strains (CBS 113027, CBS 125582) of the type species *T.vestitum* (Lutzoni et al. 2004; Spatafora et al. 2006; Vu et al. 2019); however, the phylogenetic relationships among species of this genus are unclear. A recent study on the phylogeny of Sordariomycetes has shown that *T.vestitum* is closely related to two *Phialemoniopsis* spp. (*P.endophytica* and *P.ocularis*), but their phylogenetic and taxonomic relationships have not been clarified (Dong et al. 2021; Hyde et al. 2021).

The genus *Phialemoniopsis* was placed in Phialemoniopsidaceae (Diaporthomycetidae family *incertae sedis*, Sordariomycetes; Hyde et al. 2021). Species of this genus are widely distributed in various environments and substrates, including industrial water, plant materials, raw sewage, and soil (Gams and McGinnis 1983; Halleen et al. 2007; Su et al. 2016). Several species have been reported from parts of the human body, such as blood, eye, toenail, skin, and sinus (Perdomo et al. 2013; Tsang et al. 2014), and some of them have also been isolated from patients with keratomycosis and phaeohyphomycosis (Perdomo et al. 2013; Desoubeaux et al. 2014). All species in this genus are known to be asexual.

In our ongoing taxonomic study of sordariomycetous fungi in Japan, several new specimens of *Thyridium*-like sexual morphs were collected. Single ascospore isolates from these specimens formed typical *Phialemoniopsis*-like asexual morphs in culture, suggesting that both genera are closely related. This study aims to reveal the taxonomic and phylogenetic relationships between *Thyridium* and *Phialemoniopsis*, and to clarify their ordinal position in Sordariomycetes.

## ﻿Material and method

### ﻿Isolation and morphological observation

All materials were obtained from Japan. Morphological characteristics were observed in preparations mounted in distilled water by differential interference and phase contrast microscopy (Olympus BX53) using images captured with an Olympus digital camera (DP21). All specimens were deposited in the herbarium at Hirosaki University (HHUF), Hirosaki, Japan. Single spore isolations were performed from all specimens. Colony characteristics were recorded from growth on potato dextrose agar (PDA), malt extract agar (MEA), and oatmeal agar (OA) from Becton, Dickinson and Company (MD, USA), after a week at 25 °C in the dark. Colony colours were recorded according to Rayner (1970). To observe the asexual morphs in culture, 5 mm squares of mycelial agar were placed on water agar containing sterilised plant substrates such as rice straws and banana leaves. Then these plates were incubated at 25 °C for 2 weeks in the dark. When the substrates were colonised, the plates were incubated at 25 °C under black light blue illumination for 1–2 weeks to observe sporulation.

### ﻿Phylogenetic analyses

DNA was extracted from four isolates using the ISOPLANT II kit (Nippon Gene, Tokyo, Japan) following the manufacturer’s instructions. The following loci were amplified and sequenced: the internal transcribed spacer (ITS) region with primers ITS1 and ITS4 (White et al. 1990), the large subunit nuclear ribosomal DNA (LSU) with primers LR0R (Rehner and Samuels 1994) and LR5 or LR7 (Vilgalys and Hester 1990), the second largest RNA polymerase II subunit (*rpb2*) gene with primers fRPB2-5F and fRPB2-7cR (Liu et al. 1999), the translation elongation factor 1-alpha (*tef1*) gene with primers 983F and 2218R (Rehner and Buckley 2005), the actin (*act*) gene with primers Act-1 and Act-5ra (Voigt and Wöstemeyer 2000) and the beta-tubulin (*tub2*) gene with primers TUB-F and TUB-R (Cruse et al. 2002). PCR products were purified using the FastGene Gel/PCR Extraction Kit (Nippon Gene, Tokyo, Japan) following the manufacturer’s instructions and sequenced at SolGent (South Korea). Newly generated sequences were deposited in GenBank (Table 1).

**Table 1.** Isolates and GenBank accessions of sequences used in the phylogenetic analyses of Sordariomycetes (Fig. 1).

Taxon	Isolate^a^	Status^b^	GenBank accession numbers^a^			Ref.^c^
			LSU	*rpb2*	*tef1*	
*Acrodictysaquatica*	MFLUCC 18-0356	HT	MG835712	–	–	47
*Acrodictysbambusicola*	HSAUP myr9510		KX033564	–	–	44
*Annulatascusvelatisporus*	A70 18		AY316354	–	–	3
*Annulusmagnustriseptatus*	CBS 128831		GQ996540	JQ429258	–	25, 29
*Ascitendusaustriascus*	CBS 131685		GQ996539	JQ429257	–	25, 29
*Atractosporareticulata*	CBS 127884	HT	KT991660	KT991649	–	41
*Atractosporathailandensis*	KUMCC 16-0067	HT	MF374362	MF370951	MF370962	45
*Barbatosphaeriaarboricola*	CBS 127689	HT	KM492862	KM492901	–	38
*Barbatosphaeriabarbirostris*	CBS 121149		EF577059	KM492903	–	18, 38
*Barbatosphaeriavarioseptata*	CBS 137797	HT	KM492869	KM492907	–	38
*Barrmaeliarhamnicola*	CBS 142772	ET	MF488990	MF488999	MF489009	52
*Bombardiabombarda*	AFTOL-ID 967		DQ470970	DQ470923	DQ471095	14
*Calosphaeriapulchella*	CBS 115999	IT	AY761075	GU180661	FJ238421	8, 27
*Camaropsmicrospora*	CBS 649.92		AY083821	DQ470937	–	13, 14
*Camarotellacostaricensis*	MM-149		KX430484	KX451954	KX451982	43
*Cancellidiumcinereum*	MFLUCC 18-0424	HT	MT370363	MT370486	MT370488	57
*Cancellidiumgriseonigrum*	MFLUCC 17-2117	HT	MT370364	MT370487	–	57
*Ceratolentacaudata*	CBS 125234	HT	JX066704	JX066699	–	33
	PRM 899855		JX066705	–	–	33
*Chaetosphaeriaciliata*	ICMP 18253		GU180637	GU180659	–	27
*Chaetosphaeriacurvispora*	ICMP 18255		GU180636	GU180655	–	27
*Cryptadelphiagroenendalensis*	SH12		EU528007	–	–	20
	SMH3767		EU528001	–	–	20
*Diaporthephaseolorum*	NRRL 13736		U47830	–	–	1
*Distoseptisporaobpyriformis*	MFLUCC 17-1694	HT	MG979764	MG988415	MG988422	48
*Distoseptisporarostrata*	MFLUCC 16-096	HT	MG979766	MG988417	MG988424	48
*Endoxylaoperculata*	UAMH 11085		JX460992	KY931927	–	34, 49
*Entosordariaperfidiosa*	CBS 142773	ET	MF488993	MF489003	MF489012	52
*Fluminicolaaquatica*	MFLUCC 15-0962	HT	MF374366	–	MF370960	45
*Fluminicolasaprotrophitica*	MFLUCC 15-0976	HT	MF374367	MF370954	MF370956	45
*Gnomoniagnomon*	CBS 199.53		AF408361	DQ470922	DQ471094	2, 14
*Jobellisiafraterna*	SMH2863		AY346285	–	–	4
*Jobellisialuteola*	SMH2753		AY346286	–	–	4
*Lansporacoronata*	AFTOL-ID 736		U46889	DQ470899	–	14
*Lasiosphaeriaovina*	SMH4605		AY436413	AY600284	DQ836908	6, 7, 16
*Lentomitellacirrhosa*	ICMP 15131	ET	AY761085	KM492911	–	11, 38
*Lentomitellacrinigera*	CBS 138678		KY931811	–	–	49
*Linocarponlivistonae*	HKUM 6520		DQ810205	DQ810248	–	10
*Magnaporthesalvinii*	M 21		JF414887	–	JF710406	28
*Magnaporthiopsisagrostidis*	CBS 142740	HT	KT364754	–	KT364756	37
*Melanconisstilbostoma*	CBS 109778		AF408374	EU219299	EU221886	2
*Myrmecridiummontsegurinum*	JF 13180	HT	KT991664	KT991654	–	41
*Myrmecridiumschulzeri*	CBS 100.54		EU041826	–	–	17
*Myrmecridiumthailandicum*	CBS 136551	HT	KF777222	–	–	30
*Neolinocarponenshiense*	HKUCC 2983		DQ810221	DQ810244	–	10
*Neolinocarponglobosicarpum*	HKUCC 1959		DQ810224	DQ810245	–	10
*Ophiostomapiliferum*	CBS 158.74		DQ470955	DQ470905	DQ471074	14
*Ophiostomastenoceras*	CBS 139.51		DQ836904	DQ836891	DQ836912	16
*Papulosaamerospora*	AFTOL-ID 748		DQ470950	DQ470901	DQ471069	14
*Pararamichloridiumcaricicola*	CBS 145069	HT	MK047488	–	–	46
*Pararamichloridiumlivistonae*	CBS 143166	HT	MG386084	–	–	54
*Pararamichloridiumverrucosum*	CBS 128.86	HT	MH873621	–	–	56
*Phaeoacremoniumfraxinopennsylvanica*	M.R. 3064		HQ878595	HQ878609	–	26
*Phaeoacremoniumnovae-zealandiae*	CBS 110156	HT	AY761081	–	–	8
*Phomatosporabellaminuta*	AFTOL-ID 766		FJ176857	FJ238345	–	23
*Phomatosporabiseriata*	MFLUCC 14-0832A		KX549448	–	–	51
*Phyllachoragraminis*	TH-544		KX430508	–	–	43
*Pleurostomaootheca*	CBS 115329	IT	AY761079	HQ878606	FJ238420	8, 23, 26
*Pseudostanjehughesiaaquitropica*	MFLUCC 16-0569	HT	MF077559	–	MF135655	53
*Pseudostanjehughesialignicola*	MFLUCC 15-0352	HT	MK849787	MN124534	MN194047	55
*Pyriculariaborealis*	CBS 461.65		DQ341511	–	–	24
*Pyriculariabothriochloae*	CBS 136427	HT	KF777238	–	–	30
*Rhamphoriadelicatula*	CBS 132724		FJ617561	JX066702	–	22, 33
*Rhamphoriapyriformis*	CBS 139024		MG600397	MG600401	–	50
*Rubellisphaeriaabscondita*	CBS 132078	HT	KT991666	KT991657	–	41
*Sordariafimicola*	CBS 723.96		AY780079	DQ368647	–	9, 19
*Spadicoidesbina*	CBS 137794		KY931824	KY931851	–	49
*Sporidesmiumminigelatinosa*	NN 47497		DQ408567	DQ435090	–	12
*Sporidesmiumparvum*	HKUCC 10836		DQ408558	–	–	12
*Thyridiumcornearis*	CBS 131711	HT	KJ573450	–	LC382144	36
*Thyridiumcurvatum*	CBS 490.82	HT	AB189156	–	LC382142	15
*Thyridiumendophyticum*	ACCC 38980	HT	KT799560	–	–	42
*Thyridiumflavostromatum*	**KT 3891 = MAFF 247509**	HT	**LC655963**	**LC655967**	**LC655971**	This study
*Thyridiumhongkongense*	HKU39	HT	KJ573447	–	–	36
*Thyridiumlimonesiae*	CBS 146752	HT	MW050976	–	–	58
*Thyridiumoculorum*	CBS 110031	HT	KJ573449	–	LC382145	36
*Thyridiumpluriloculosum*	CBS 131712	HT	HE599271	–	LC382141	32
	**KT 3803 = MAFF 247508**		**LC655964**	**LC655968**	**LC655972**	This study
*Thyridiumpunctulatum*	**KT 1015 = MAFF 239669**		**LC655965**	**LC655969**	**LC655973**	This study
	**KT 3905 = MAFF 247510**	ET	**LC655966**	**LC655970**	**LC655974**	This study
*Thyridiumvestitum*	CBS 113027		AY544671	DQ470890	DQ471058 ^d^	5, 14
	CBS 125582		MH875182	–	–	56
*Tirisporellabeccariana*	BCC 36737		JQ655450	–	–	39
*Tirisporellabisetulosus*	BCC 00018		EF622230	–	–	21
*Wongiagriffinii*	BRIP 60377		KU850470	–	KU850466	40
*Woswasiaatropurpurea*	CBS 133167	HT	JX233658	JX233659	–	31
*Xylochrysislucida*	CBS 135996	HT	KF539911	KF539913	–	35
*Xylolentiabrunneola*	PRA-13611	HT	MG600398	MG600402	–	50

^a^ Strains and sequences generated in this study are shown in **bold**. ^b^ET = epitype; HT = holotype; IT = isotype ^c^ 1: Viljoen et al. 1999; 2: Castlebury et al. 2002; 3: Raja et al. 2003; 4: Huhndorf et al. 2004; 5: Lutzoni et al. 2004; 6: Miller and Huhndorf 2004a; 7: Miller and Huhndorf 2004b; 8: Réblová et al. 2004; 9: Miller and Huhndorf 2005; 10: Bahl 2006; 11: Réblová 2006; 12: Shenoy et al. 2006; 13: Smith et al. 2006; 14: Spatafora et al. 2006; 15: Yaguchi et al. 2006; 16: Zhang et al. 2006; 17: Arzanlou et al. 2007; 18: Réblová 2007; 19: Tang et al. 2007; 20: Huhndorf et al. 2008; 21: Pinruan et al. 2008; 22: Réblová 2009; 23: Schoch et al. 2009; 24: Thongkantha et al. 2009; 25: Réblová et al. 2010; 26: Réblová 2011; 27: Réblová et al. 2011; 28: Zhang et al. 2011; 29: Réblová et al. 2012; 30: Crous et al. 2013; 31: Jaklitsch et al. 2013; 32: Perdomo et al. 2013; 33: Réblová 2013; 34: Untereiner et al. 2013; 35: Réblová et al. 2014; 36: Tsang et al. 2014; 37: Crous et al. 2015b; 38: Réblová et al. 2015; 39: Suetrong et al. 2015; 40: Khemmuk et al. 2016; 41: Réblová et al. 2016; 42: Su et al. 2016; 43: Mardones et al. 2017; 44: Xia et al. 2017; 45: Zhang et al. 2017; 46: Crous et al. 2018; 47: Hyde et al. 2018; 48: Luo et al. 2018; 49: Réblová et al. 2018; 50: Réblová and Štěpánek 2018; 51: Senanayake et al. 2018; 52: Voglmayr et al. 2018; 53: Yang et al. 2018; 54: Crous et al. 2019; 55: Luo et al. 2019; 56: Vu et al. 2019; 57: Hyde et al. 2021; 58: Martinez et al. 2021. ^d^ This tef1 sequence (DQ471058) of *Thyridiumvestitum* was excluded from this analysis. A Blast search using this sequence suggested that it is close to *Phialemoniumobovatum* (Cephalothecales) rather than *Thyridium/Phialemoniopsis* (Thyridiales).

Primary analysis of LSU-*rpb2*-*tef1* sequences from 88 strains of Sordariomycetes (Table 1) was conducted to clarify the ordinal/familial placement of *Thyridium* (or *Phialemoniopsis*) species. *Barrmaeliarhamnicola* and *Entosordariaperfidiosa* (Xylariomycetidae) were used as outgroups. As a secondary analysis, single gene trees of ITS, *act* and *tub2*, and a combined tree of these three loci were generated to assess the species boundaries of 17 strains within *Thyridium*/*Phialemoniopsis* (Table 2). All sequence alignments (LSU, ITS, *rpb2*, *tef1*, *act* and *tub2*) were produced using the server version of MAFFT (http://www.ebi.ac.uk/Tools/msa/mafft), checked and refined using MEGA v. 7.0 (Kumar et al. 2016).

**Table 2.** Isolates and GenBank accessions of sequences used in the phylogenetic analyses of *Thyridiu*m species (Fig. 2).

Taxon	Isolate^a^	Substrate/Host	Status^b^	GenBank accession numbers^a^			Ref.^c^
				ITS	*act*	*tub2*	
*Thyridiumcornearis*	CBS 131711	human corneal fluid	HT	KJ573445	HE599252	HE599301	1, 2
	UTHSC 06-1465	shin aspirate		HE599285	HE599253	HE599302	2
*Thyridiumcurvatum*	CBS 490.82	skin lesion	HT	AB278180	HE599258	HE599307	2
	UTHSC R-3447	human eye		HE599291	HE599259	HE599308	2
*Thyridiumendophyticum*	ACCC 38979	lower stem of *Luffacylindrica* (endophyte)		KT799556	KT799553	KT799562	4
	ACCC 38980	lower stem of *Luffacylindrica* (endophyte)	HT	KT799557	KT799554	KT799563	4
*Thyridiumflavostromatum*	**KT 3891 = MAFF 247509**	dead twigs of *Phyllostachyspubescens*	HT	**LC655959**	**LC655979**	**LC655975**	This study
*Thyridiumhongkongense*	HKU39	the right forearm nodule biopsy of a human	HT	KJ573442	KJ573452	KJ573457	3
*Thyridiumlimonesiae*	CBS 146752	Skin nodule	HT	MW050977	MW349126	MW048608	6
*Thyridiumoculorum*	CBS 110031	human keratitis	HT	KJ573444	HE599247	HE599296	2, 3
	UTHSC 05-2527	peritoneal dialysis catheter		HE599281	HE599249	HE599298	2
*Thyridiumpluriloculosum*	CBS 131712	human toe nail	HT	HE599286	HE599254	HE599303	2
	**KT 3803 = MAFF 247508**	dead wood of *Betulamaximowicziana*	HT	**LC655960**	**LC655980**	**LC655976**	This study
	UTHSC 09-3589	synovial fluid		HE599287	HE599255	HE599304	2
*Thyridiumpunctulatum*	**KT 1015 = MAFF 239669**	dead culms of *Phyllostachyspubescens*		**LC655961**	**LC655981**	**LC655977**	This study
	**KT 3905 = MAFF 247510**	dead twigs of Phyllostachysnigravar.nigra	ET	**LC655962**	**LC655982**	**LC655978**	This study
*Thyridiumvestitum*	CBS 125582			MH863721	–	–	5

^a^ Strains and sequences generated in this study are shown in **bold**. ^b^ET = epitype; HT = holotype ^c^ 1: Tang et al. 2007; 2: Perdomo et al. 2013; 3: Tsang et al. 2014; 4: Su et al. 2016; 5: Vu et al. 2019; 6: Martinez et al. 2021.

Phylogenetic analyses were conducted using maximum-likelihood (ML) and Bayesian methods. The optimum substitution models for each dataset were estimated using Kakusan4 software (Tanabe 2011) based on the Akaike information criterion (AIC; Akaike 1974) for ML analysis and the Bayesian information criterion (BIC; Schwarz 1978) for Bayesian analysis. ML analyses were performed using the TreeFinder Mar 2011 program (http://www.treefinder.de) based on the models selected with the AICc4 parameter (used sequence length as sample size). ML bootstrap support (ML BS) values were obtained using 1000 bootstrap replicates. Bayesian analyses were performed using MrBayes v. 3.2.6 (Ronquist et al. 2012), with substitution models selected based on the BIC4 parameter (used sequence length as sample size). Two simultaneous and independent Metropolis-coupled Markov chain Monte Carlo (MCMC) runs were performed for 9,000,000 generations for primary analysis and 1,000,000 generations for secondary analyses (except for the ITS dataset for 1,500,000 generations) with the tree sampled every 1,000 generations. Convergence of the MCMC procedure was assessed from the effective sample size scores (all > 100) using MrBayes and Tracer v. 1.6 (Rambaut et al. 2014). First 25% of the trees were discarded as burn-in, and the remainder were used to calculate the 50% majority-rule trees and to determine the posterior probabilities (PPs) for individual branches. These alignments were submitted to TreeBASE under study number S28934.

## ﻿Result

### ﻿Phylogeny

**Figure 1.**
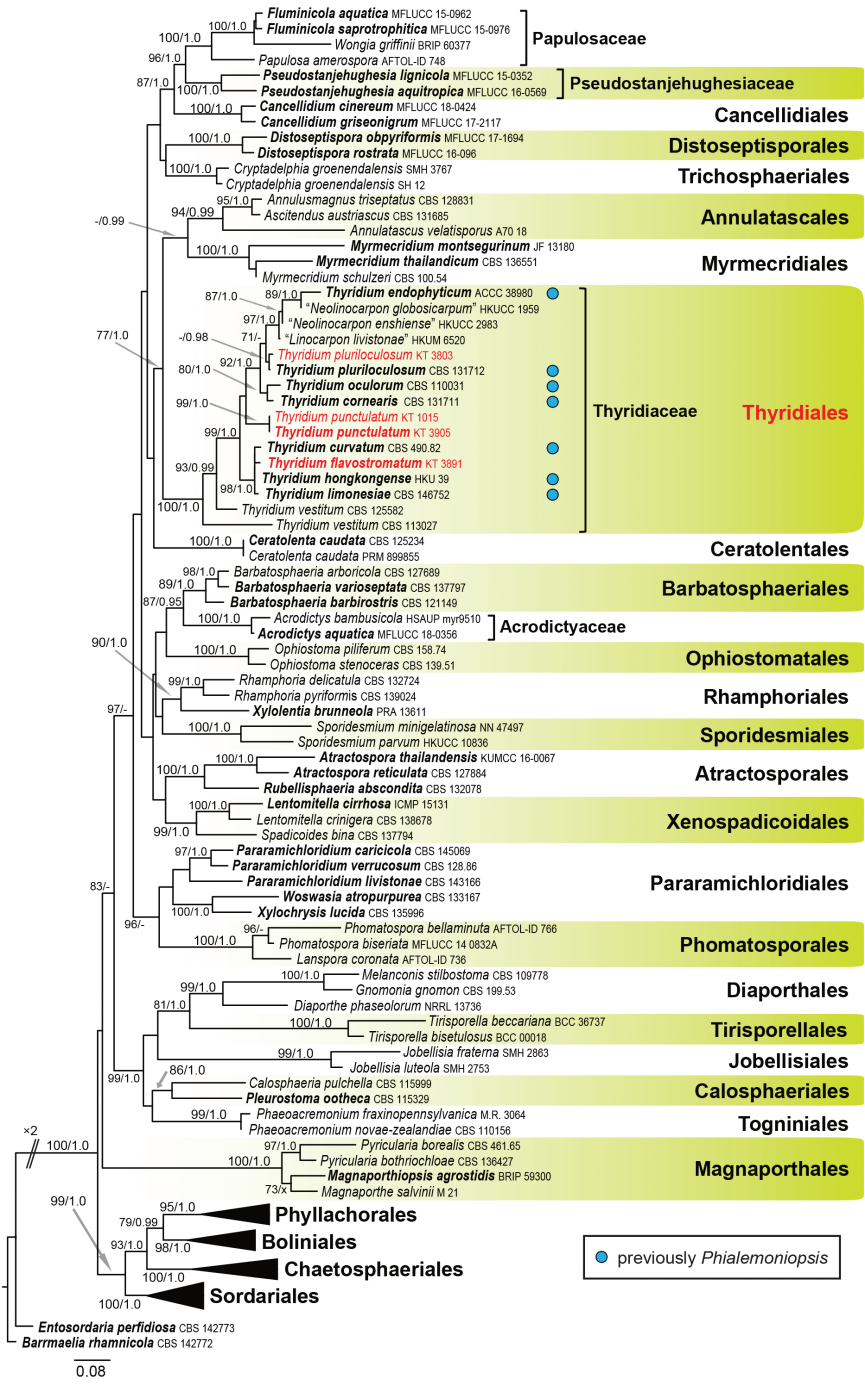
Maximum-likelihood tree of Sordariomycetes based on combined LSU, *rpb2* and *tef1* sequence. ML bootstrap proportion (BP) greater than 70% and Bayesian posterior probabilities (PP) above 0.95 are presented at the nodes as MLBP/Bayesian PP and a node not present in the Bayesian analysis is shown with ‘x’. A hyphen (‘-’) indicates values lower than 70% BP or 0.95 PP. Ex-holotype, isotype, paratype and epitype strains are shown in bold and the newly obtained sequences are shown in red. Strains previously described as *Phialemoniopsis* species are marked with a blue circle. The scale bar represents nucleotide substitutions per site.

For primary analysis, ML and Bayesian phylogenetic trees were generated using an aligned sequence dataset comprising of LSU (1,205 base pairs), *rpb2* (1,059 bp) and *tef1* (954 bp). Of the 3,218 characters included in the alignment, 1,478 were variable and 1,686 were conserved. This combined dataset provided higher confidence values for ordinal and familial classification than those of individual gene trees, with 25 orders and three families (order unknown) being reconstructed in Sordariomycetes (Fig. 1). ML analysis of the combined dataset was conducted based on the selected substitution model for each partition (GTR+G for LSU, J2+G for the first and third codon positions of *rpb2*, J1+G for the second codon positions of *rpb2*, F81+G for the first codon positions of *tef1*, JC69+G for the second codon positions of *tef1*, and J2+G for the third codon position of *tef1*). The ML tree with the highest log likelihood (–43687.562) is shown in Fig. 1. Topology recovered by Bayesian analysis was almost identical to that of the ML tree. All species previously described as *Phialemoniopsis* (marked with blue circle in Fig. 1), one species of “*Linocarpon*”, two species of “*Neolinocarpon*” and four strains newly obtained in this study formed a monophyletic clade with the type species of *Thyridium* (*T.vestitum*). Their monophyly was completely supported (100% ML BS/1.0 Bayesian PP; Fig. 1). The family Thyridiaceae was found to be related to Annulatascales and Myrmecridiales but did not cluster with any existing order in Sordariomycetes.

**Figure 2.**
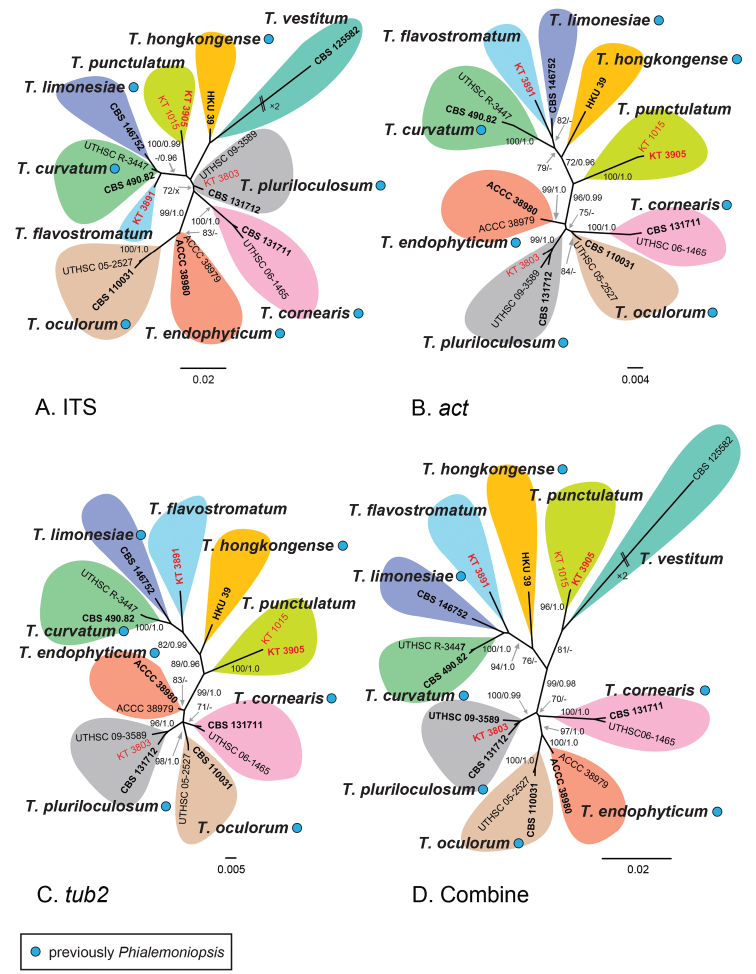
Maximum-likelihood tree of *Thyridium* species based on each ITS (A), *act* (B), *tub2* (C) and combined sequences (ITS-*act*-*tub2*; D). ML bootstrap proportion (BP) greater than 70% and Bayesian posterior probabilities (PP) above 0.95 are presented at the nodes as MLBP/Bayesian PP. A hyphen (‘-’) indicates values lower than 70% BP or 0.95 PP and a node not present in the Bayesian analysis is shown with ‘x’. Ex-holotype and epitype strains are shown in bold and the newly obtained sequences are shown in red. Strains previously as *Phialemoniopsis* species are marked with a blue circle. The scale bars represent nucleotide substitutions per site.

For secondary analysis, ML and Bayesian phylogenetic trees were generated using sequences of ITS (483 bp), *act* (646 bp), *tub2* (375 bp), and a combined dataset of these three regions (1,504 bp). The selected substitution models for each region were as follows: J2ef+G for ITS, F81+H for the first and second codon positions of *act*, J2+G for the third codon position of *act*, K80+H for the first codon positions of *tub2*, JC69+H for the second codon position of *tub2* and TN93+H for the third codon position of *tub2*. The ML trees with the highest log likelihood (–1172.0198 in ITS, –1196.6012 in *act*, –859.37115 in *tub2* and –3315.7254 in ITS-*act*-*tub2*) are shown in Fig. 2. Our results confirmed close phylogenetic relationships between *Thyridium* and *Phialemoniopsis* (Fig. 2A–D). Except for *act* (Fig. 2B) and *tub2* (Fig. 2C), where sequence data of *T.vestitum* were unavailable, the existence of ten distinct species was suggested (Fig. 2A, D). The following three lineages were found in our four strains (Fig. 2A–D): 1) a bambusicolous lineage (KT 3891) close to *T.curvatum* and *T.limonesiae*, 2) a fungus on *Betulamaximowicziana* (KT 3803) nested with *T.pluriloculosum*, which was previously reported from clinical sources (Perdomo et al. 2013), and 3) another bambusicolous lineage represented by two strains (KT 1015 and KT 3905).

### ﻿Taxonomy

A new order, Thyridiales, is introduced to accommodate Thyridiaceae because its lineage is phylogenetically and morphologically distinct from any known orders in Sordariomycetes. We concluded *Thyridium* and *Phialemoniopsis* to be congeneric based on their morphological similarities and phylogenetic relatedness. An expanded generic circumscription of *Thyridium* that integrates the generic concept of *Phialemoniopsis* is provided below. One new species and eight new combinations of *Thyridium* are proposed.

#### ﻿ Thyridiales

Taxon classificationFungiThyridialesThyridiaceae

R. Sugita & Kaz. Tanaka ord. nov.

0B63AD08-AECC-5B79-B91B-F3E274CC1C22

841916

##### Type family.

Thyridiaceae J.Z. Yue & O.E. Erikss., Syst. Ascom. 6(2): 233 (1987).

##### Sexual morph.

Stromata scattered to grouped. Ascomata perithecial, subglobose to ampulliform. Ostiolar neck cylindrical, periphysate. Paraphyses numerous, unbranched, cylindrical, hyaline. Asci unitunicate, cylindrical, with an apical annulus, pedicellate. Ascospores obovoid to ellipsoid, muriform, hyaline to brown.

##### Asexual morph.

Coelomycetous asexual morph: Conidiomata pycnidial, globose to subglobose. Conidiogenous cells phialidic. Conidia ellipsoidal to obovoid, aseptate, hyaline. Hyphomycetous synasexual morph: Colonies effuse or sporodochial. Conidiophores micronematous, mononematous, simple or branched, hyaline, thin-walled. Conidiogenous cells phialidic. Conidia ellipsoidal to allantoid, aseptate, hyaline.

##### Notes.

Thyridiaceae has been treated as *incertae sedis* in Sordariomycetes (Yue and Eriksson 1987). Members of Thyridiaceae differ from Myrmecridiales by having pycnidial conidiomata, becoming cup-shaped in the coelomycetous state and micronematous conidiophores with monophialidic conidiogenous cells in the hyphomycetous state. Myrmecridiales have brown thick-walled conidiophores with polyblastic conidiogenous cells (Crous et al. 2015a). Annulatascales have relatively massive refractive, well-developed, conspicuous apical annulus in asci (Wong et al. 1999; Campbell and Shearer 2004; Dong et al. 2021). In contrast, those of members of Thyridiaceae are compact and inconspicuous. Therefore, a new order, Thyridiales, is introduced for this lineage.

#### ﻿ Thyridiaceae

Taxon classificationFungiThyridialesThyridiaceae

J.Z. Yue & O.E. Erikss., Syst. Ascom. 6(2): 233 (1987).

E3210D80-91C5-5456-9636-F9F6B82A3631

Phialemoniopsidaceae K.D. Hyde & Hongsanan, [as Phialemoniopsaceae] Fungal Divers. 107: 95 (2021).

##### Type genus.

*Thyridium* Nitschke, Pyrenomyc. Germ. 1: 110 (1867).

##### Notes.

Phialemoniopsidaceae is considered a synonym of Thyridiaceae because *Phialemoniopsis*, the type genus of Phialemoniopsidaceae, was revealed congeneric with *Thyridium* and is placed in the synonymy of the latter genus in this study. The type genera of both families, that is, *Thyridium* and *Phialemoniopsis*, share many morphological features in their asexual states, as noted below.

#### ﻿ Thyridium

Taxon classificationFungiThyridialesThyridiaceae

Nitschke, Pyrenomyc. Germ. 1: 110 (1867).

EAA62308-9273-51E9-B4F6-0223F0BBF041

Melanospora subgen. Bivonella Sacc., Syll. fung. (Abellini) 2: 464 (1883).
Bivonella (Sacc.) Sacc., Syll. fung. (Abellini) 9: 989 (1891).
Pleurocytospora Petr., Annls mycol. 21: 256 (1923).
Sinosphaeria J.Z. Yue & O.E. Erikss., Syst. Ascom. 6: 231 (1987).
Phialemoniopsis Perdomo, Dania García, Gené, Cano & Guarro, Mycologia 105: 408 (2013).

##### Type species.

*Thyridiumvestitum* (Fr.) Fuckel, Jb. nassau.Ver. Naturk. 23–24: 195 (1870) [1869–70].

##### Sexual morph.

Stromata scattered to grouped, subepidermal to erumpent, yellowish to dark brown, red in KOH or not changing. Ascomata perithecial, subglobose to ampulliform, single to grouped, immersed in stromata to erumpent through host surface. Ascomatal wall composed of several layers of polygonal, dark brown cells. Ostiolar neck cylindrical, short or long, separated or convergent in upper stromata, periphysate. Paraphyses numerous, septate, unbranched, cylindrical, hyaline. Asci unitunicate, cylindrical, broadly rounded at the apex, with a pronounced non-amyloid apical annulus, pedicellate. Ascospores obovoid or ellipsoid, smooth, pale brown to brown, with several transverse and 0–3 longitudinal or oblique septa.

##### Asexual morph.

Coelomycetous and/or hyphomycetous morphs formed. Coelomycetous asexual morph: Conidiomata pycnidial, single to grouped, superficial or immersed in stromata, globose to subglobose, composed of polygonal to prismatic cells, often becoming cup-shaped when mature, surrounded by setose hyphae. Conidiomatal wall composed of several layers of polygonal, dark brown cells. Ostiolar neck cylindrical, central, periphysate. Setose hyphae erect, usually unbranched, septate, cylindrical, with slightly pointed or blunt tips, hyaline to pale brown, smooth-walled. Conidiophores hyaline, thin-walled, simple or irregularly branched, with branches bearing a small group of phialides terminally. Phialides swollen at the base, tapering at the tip, hyaline. Conidia obovoid to oblong, with a slightly apiculate base, hyaline, smooth-walled, in slimy masses. Hyphomycetous synasexual morph: Colonies effuse or sporodochial. Conidiophores micronematous, mononematous, hyaline, thin-walled, simple or irregularly branched, with branches bearing a small group of phialides terminally. Phialides swollen at the base, tapering at the tip, hyaline. Adelophialides absent or rarely present. Conidia ellipsoidal to allantoid, with a slightly apiculate base, hyaline, smooth-walled, in slimy head. Chlamydospores absent or rarely present, hyaline to pale brown, thick- and rough-walled.

##### Notes.

The newly obtained *Thyridium* collections formed synasexual morphs, coelomycetous and hyphomycetous, in culture that were similar to those of *Phialemoniopsis*, having coelomycetous and/or hyphomycetous conidial states in culture (Perdomo et al. 2013). In this study, *Phialemoniopsis* is treated as a synonym of *Thyridium* because of their morphological similarities in asexual morphs and phylogenetic relatedness. The genus *Pleurocytospora* has been proposed as a synonym of *Thyridium* by culture studies (Leuchtmann and Müller 1986). We agree that the morphological features of *Pleurocytospora*, such as phialidic conidiogenous cells and hyaline, ellipsoidal conidia formed from both coelomycetous and hyphomycetous states (Leuchtmann and Müller 1986), are almost identical to those of the generic concept of *Thyridium* emended here.

We accept both *Bivonella* and *Sinosphaeria* as synonyms of *Thyridium*, as proposed in previous studies (Eriksson and Yue 1989; Checa et al. 2013). *Sinosphaeria* (typified by *S.bambusicola* = *Thyridiumchrysomallum*; Yue and Eriksson 1987) was established as a new genus without knowing the existence of *Bivonella* (typified by *B.lycopersici*; Saccardo 1891). Both genera are characterised by yellowish stromata. The validity of these genera being synonymised under *Thyridium* is confirmed by the presence of *T.flavostromatum*, which has yellowish stromata, in the strongly supported *Thyridium* clade (Fig. 1).

**Figure 3.**
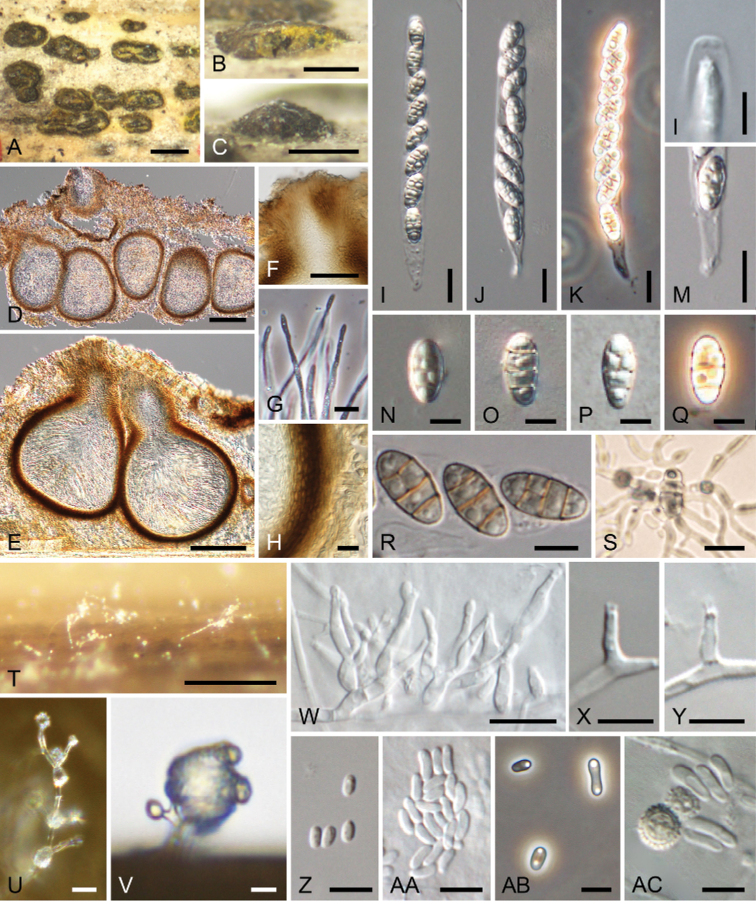
*Thyridiumflavostromatum* (**A–S** KT 3891 = HHUF 30647 **T–AC** culture KT 3891 = MAFF 247509) **A–S** sexual morph **A–C** appearance of stromata on substrate **D, E** ascomata in longitudinal section (**D** in 2% KOH) **F** ostiolar neck of ascoma **G** paraphyses **H** ascomatal wall **I–K** asci **L** apex of the ascus **M** stipe of the ascus **N–R** ascospores **S** germinating ascospore **T–AC** hyphomycetous asexual morph **T** sporulation in culture **U** phialides **V** slimy conidial heads **W** conidiophores **X** phialide **Y** adelophialide **Z–AB** conidia **AC** chlamydospores and conidia. Scale bars: 1 mm (**A**); 500 µm (**B, C**); 100 µm (**D, E**); 50 µm (**F**); 10 µm (**G–K, M, S, U, V**); 5 µm (**L, N–R, W–AC**); 250 µm (**T**).

#### ﻿ Thyridium flavostromatum

Taxon classificationFungiThyridialesThyridiaceae

R. Sugita & Kaz. Tanaka sp. nov.

07C7BEFD-8883-54AB-B333-04BC7976E268

841917

Figs 3 , 6A

##### Holotype.

Japan, Yamaguchi, Nagato, Misumikami, near Kusaritoge, on dead twigs of *Phyllostachyspubescens*, 26 March 2018, K. Tanaka, K. Arayama and R. Siguta, KT 3891 (HHUF 30647, holotype designated here), living culture MAFF 247509.

##### Etymology.

The name refers to yellowish stromata.

##### Sexual morph.

Stromata scattered to grouped, subepidermal, becoming erumpent to superficial, 0.7–1.4 mm long, 0.4–0.7 mm wide, yellowish to dark brown, red in 2% KOH. Ascomata perithecial, subglobose to ampulliform, mostly 2–6 grouped, 190–240 µm high, 200–220 µm diam., immersed in stromata to erumpent through host surface. Ascomatal wall 15–23 µm thick, composed of 5–8 layers of polygonal, 2.5–7 × 1.5–3.5 µm, dark brown cells. Ostiolar neck central, cylindrical, 80–140 µm long, 55–90 µm wide, periphysate. Paraphyses numerous, septate, unbranched, cylindrical, 50–105 µm long. Asci unitunicate, cylindrical, 62.5–90 × 6.5–10 µm (av. 78.7 × 7.8 µm, n = 30), broadly rounded at the apex, with a pronounced non-amyloid apical annulus, short-stalked (5–17.5 µm long), with 8 ascospores. Ascospores obovoid to ellipsoid, smooth, hyaline to pale brown, with 3 transverse and 0–2 vertical septa, 9.5–14 × 5–7.5 µm (av. 11.3 × 5.8 µm, n = 50), l/w 1.4–2.5 (av. 2.0, n = 50).

##### Asexual morph (nature).

Not observed.

##### Asexual morph (culture).

Hyphomycetous asexual morph formed. Conidiophores micronematous, mononematous, hyaline, thin-walled, simple or irregularly branched, with branches bearing a group of 2–3 phialides terminally. Phialides swollen at the base, tapering at the tip, hyaline, 3–6 × 1–1.5 µm. Adelophialides rarely present. Conidia ellipsoidal to allantoid, with a slightly apiculate base, hyaline, smooth-walled, 2–7 × 1–2.5 µm (av. 4.1 × 1.6 µm, n = 50). Chlamydospores rarely present, solitary, 3.5–6.5 µm diam., hyaline to pale brown, thick- and rough-walled.

##### Culture characteristics.

Colonies on MEA at 25 °C attained 28–29 mm diam. after a week in the dark, whitish. On OA attained 35–37 mm diam., whitish. On PDA attained 28–31 mm diam., whitish to buff (45; Rayner 1970) (Fig. 6A).

##### Notes.

Phylogenetic analyses based on ITS, *act*, and *tub2* sequences suggested that *T.flavostromatum* was closely related to *T.curvatum*, *T.hongokgense* and *T.limonesiae* (Fig. 2), of which only *T.hongokgense* has unknown conidial state. Although *T.curvatum* forms sporodochial conidiomata (Perdomo et al. 2013), those are not found in *T.flavostromatum*. Conidia of *T.limonesiae* (2.3–4.9 × 1.4–2 μm; Martinez et al. 2021) are smaller than those of *T.flavostromatum* (2–7 × 1–2.5 µm). *Thyridiumflavostromatum* is similar to *T.lasiacidis* on *Lasiacisligulata* (Samuels and Rogerson 1989) in 1) having yellowish stromata becoming red in KOH, and 2) ellipsoidal ascospores with three transverse septa, with or without one longitudinal septum in 1–2 median cells. However, *T.lasiacidis* differs from *T.flavostromatum* by ascomata with a longer ostiolar neck (90–170 µm long) and dark brown ascospores with terminal pale brown cells (Samuels and Rogerson 1989).

#### ﻿ Thyridium pluriloculosum

Taxon classificationFungiThyridialesThyridiaceae

(Perdomo, Dania García, Gené, Cano & Guarro) R. Sugita & Kaz. Tanaka comb. nov.

8E8EB860-5BC9-58AA-BED9-2440D0F7093B

841918

Figs 4 , 6B

##### Basionym.

*Phialemoniopsispluriloculosa* Perdomo, Dania García, Gené, Cano & Guarro, Mycologia 105: 412 (2013).

##### Holotype.

USA, Nevada, human toe nail, D.A. Sutton, CBS H-20782, living culture CBS 131712 = UTHSC 04–7 = FMR 11070 (not seen).

##### Sexual morph.

Stromata scattered to grouped, pulvinate, circular to elliptical in outline, elevated beyond bark surface forming pustules, 0.6–0.7 mm high, 0.9–1.0 mm diam., dark brown to black. Ascomata perithecial, subglobose to ampulliform, 4–8 grouped, 700–780 µm high, 220–280 µm diam., immersed in stromata. Ascomatal wall 17–25 µm thick, composed of 7–10 layers of polygonal, 4–6.5 × 2–4 µm, dark brown cells. Ostiolar neck central, cylindrical, 400–430 µm long, 100–110 µm wide, periphysate. Paraphyses septate, unbranched, cylindrical, 92.5–110 µm long, 3.5–5.5 µm wide. Asci unitunicate, cylindrical, 110–175 × 9–12.5 µm (av. 145.6 × 10.3 µm, n = 15), broadly rounded at the apex, with a pronounced non-amyloid apical annulus, pedicellate (12.5–27.5 µm long), with 8 ascospores. Ascospores fusiform to ellipsoid, smooth, brown, with 3 transverse and 0–2 oblique or vertical septa, 13.5–18 × 6–8 µm (av. 15.5 × 7.3 µm, n = 50), l/w 1.7–2.6 (av. 2.1, n = 50).

##### Asexual morph (nature).

Conidiomata pycnidial, globose to subglobose, grouped, 220–300 µm high, 90–150 µm diam., immersed in stromata. Conidiomatal wall 8–18 µm thick, composed of 3–5 layers of polygonal, 3–4.5 × 2.5–4 µm, dark brown cells. Ostiolar neck central, cylindrical, 80–110 µm long, 90–110 µm wide, composed of polygonal cells, periphysate. Conidiophores hyaline, thin-walled, with branches bearing a group of 2–5 phialides terminally. Phialides tapering toward the tip, hyaline, 11–16 × 1–2 µm. Conidia ellipsoidal, with a slightly apiculate base, hyaline, smooth-walled, 3–4.5 × 1–2 µm (av. 3.7 × 1.5 µm, n = 50). Chlamydospores not observed.

**Figure 4.**
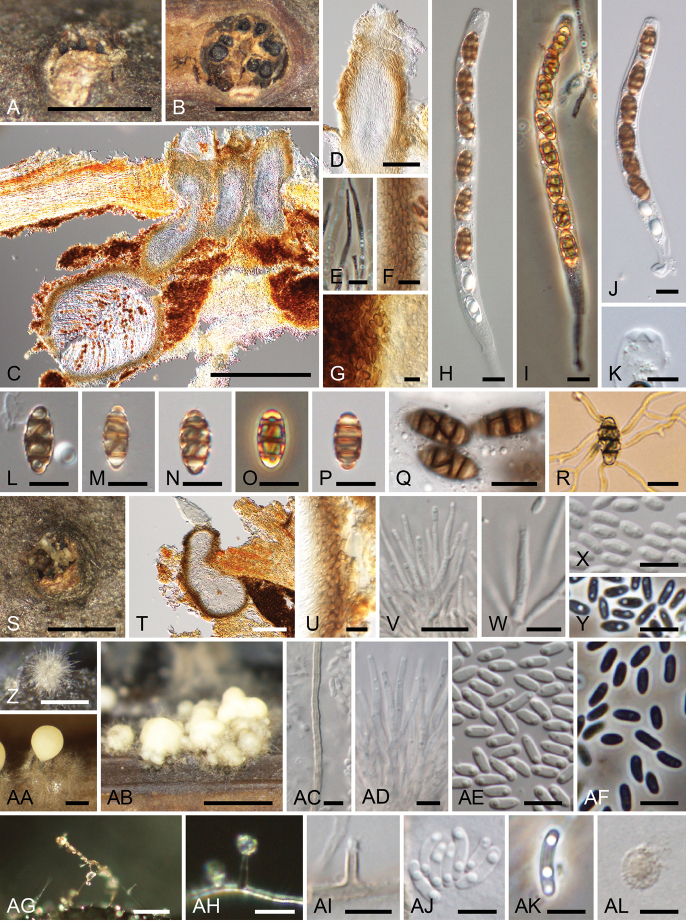
*Thyridiumpluriloculosum* (**A–Y** KT 3803 = HHUF 30648 **Z–AL** culture KT 3803 = MAFF 247508) **A–R** sexual morph **A, B** appearance of stromata on substrate (**B** transverse sections) **C** ascomata in longitudinal section **D** ostiolar neck of ascoma **E** paraphyses **F** ascomatal wall **G** pseudostromatic tissue **H–J** asci **K** apex of ascus **L–Q** ascospores **R** germinating ascospore **S–AF** coelomycetous asexual morph (**S–Y** nature **Z–AF** culture) **S** appearance of conidiomata on substrate **T** conidiomata in longitudinal section **U** conidiomatal wall **V** conidiophores **W** phialide **X, Y** conidia **Z–AB** conidiomata in culture (**AB** multiloculate conidiomata) **AC** setose hypha of conidiomata **AD** conidiophores with groups of phialides **AE, AF** conidia **AG–AL** hyphomycetous synasexual morph **AG, AH** sporulation in culture **AI** phialide **AJ, AK** conidia **AL** chlamydospores. Scale bars: 1 mm (**A, B, S, AB**); 500 µm (**C, Z, AA**); 100 µm (**D, T**); 20 µm (**AG, AH**); 10 µm (**E–J, L–R, U, V**); 5 µm (**K, W–Y, AC–AF, AI–AL**).

##### Asexual morph (culture).

Coelomycetous asexual morph: Conidiomata pycnidial, scattered, single to grouped, superficial, globose to subglobose, 180–380 µm high, mostly 80–580 µm diam., up to 1170 µm diam. when grouped, often becoming cup-shaped when mature, surrounded by setose hyphae. Conidiomatal wall composed of polygonal to prismatic, 3–4.5 × 2.5–4 µm, dark brown cells. Setose hyphae erect, usually unbranched, septate, up to 360 µm long, 2–3 µm wide, pale brown. Conidiophores hyaline, thin-walled, simple or irregularly branched, with branches bearing a group of 2–5 phialides terminally. Phialides tapering toward the tip, hyaline, 10–25 × 1–2.5 µm. Conidia ellipsoidal, with a slightly apiculate base, hyaline, smooth-walled, in slimy masses, 3–4.5 × 1–2 µm (av. 3.8 × 1.4 µm, n = 50). Hyphomycetous synasexual morph: Conidiophores micronematous, mononematous, hyaline, simple or rarely branched. Phialides slightly tapering toward the tip, 4–11 × 1–2.5 µm, hyaline. Adelophialide absent. Conidia allantoid, hyaline, smooth-walled, in slimy heads, 3–9 × 1–2.5 µm (av. 6.2 × 1.7 µm, n = 50). Chlamydospores rarely present, solitary, 3.5–6.5 µm diam., hyaline to pale brown, thick- and rough-walled.

##### Culture characteristics.

Colonies on MEA at 25 °C attained 31–33 mm diam. after a week in the dark, whitish. On OA attained 32–36 mm diam., whitish to grey olivaceous (107). On PDA attained 32–33 mm diam., whitish to buff (45) (Fig. 6B).

##### Specimen examined.

Japan, Aomori, Hirakawa, Hirofune, Shigabo Forest Park, on dead twigs of *Betulamaximowicziana*, 10 October 2017, K. Tanaka, KT 3803 (HHUF 30648), living culture MAFF 247508.

##### Notes.

The conidia from aerial hyphae of strain KT 3803 were larger (3–9 × 1–2.5 µm) in culture than those of the original description of *Thyridiumpluriloculosum* (3–5 × 1–2.5 µm; Perdomo et al. 2013). However, we identified this new collection on *Betulamaximowicziana* as *T.pluriloculosum*, based on the high sequence homology of three loci with ex-type culture of this species (CBS 131712; 99.6% in ITS, 99.2% in *act*, and 99.5% in *tub2*). The sexual-asexual relationship of *T.pluriloculosum* was verified in this study. Although this species has been reported from clinical sources as an asexual morph (Perdomo et al. 2013), the recently collected material represents a sexual morph on plant material.

In *Thyridium*, *T.betulae* has also been recorded on *Betula* sp. in France (Roumeguère 1891). Although sequences of *T.betulae* are unavailable for molecular comparison, it is clearly different from *T.pluriloculosum* in having ascospores with 5–7 transverse and one longitudinal septum.

#### ﻿ Thyridium punctulatum

Taxon classificationFungiThyridialesThyridiaceae

(I. Hino & Katum.) R. Sugita & Kaz. Tanaka comb. nov.

78E8C592-35BB-5E7A-9B95-F58E6C7079D2

841919

Figs 5 , 6C

##### Basionym.

*Pleosporapunctulata* I. Hino & Katum., *Icones Fungorum Bamb*. *Jpn.*: 181 (1961).

##### Holotype.

Japan, Shizuoka, Fuji Bamboo Garden, on dead twigs of Phyllostachysnigravar.henonis, 1 April 1958, K. Katumoto, YAM 21851.

##### Epitype.

Japan, Yamaguchi, Hagi, Akiragi, near Chikurindoro-park, on dead twigs of Phyllostachysnigravar.nigra, 26 March 2018, K. Tanaka, K. Arayama and R. Sugita, KT 3905 (HHUF 30649 epitype designated here; MBT 10004137), ex-epitype culture MAFF 247510.

##### Sexual morph.

Stromata scattered to grouped, subepidermal, becoming erumpent to superficial, 0.5–1.2 mm long, 0.2–0.4 mm wide, dark brown. Ascomata perithecial, subglobose to conical, single to 2–3 grouped, 130–190 µm high, 140–230 µm diam., immersed in stromata to erumpent through host surface. Ascomatal wall 7–15 µm thick, composed of 3–5 layers of polygonal, 3–6.5 × 1–4.5 µm, dark brown cells. Ostiolar neck central, cylindrical, 37–85 µm long, 37–63 µm wide, periphysate. Paraphyses numerous, septate, unbranched, cylindrical, hyaline, 77–103 µm long. Asci unitunicate, cylindrical, 67.5–105 × 7.5–11.5 µm (av. 82.9 × 9.4 µm, n = 60), broadly rounded at the apex, with a pronounced non-amyloid apical annulus, short-stalked (3.5–11.5 µm long), with 8 ascospores. Ascospores ellipsoid to oblong, smooth, pale brown, with 3 transverse and 1–2 vertical septa, 10–15 × 5–9 µm (av. 12.8 × 7.0 µm, n = 60), l/w 1.4–2.4 (av. 1.8, n = 60).

**Figure 5.**
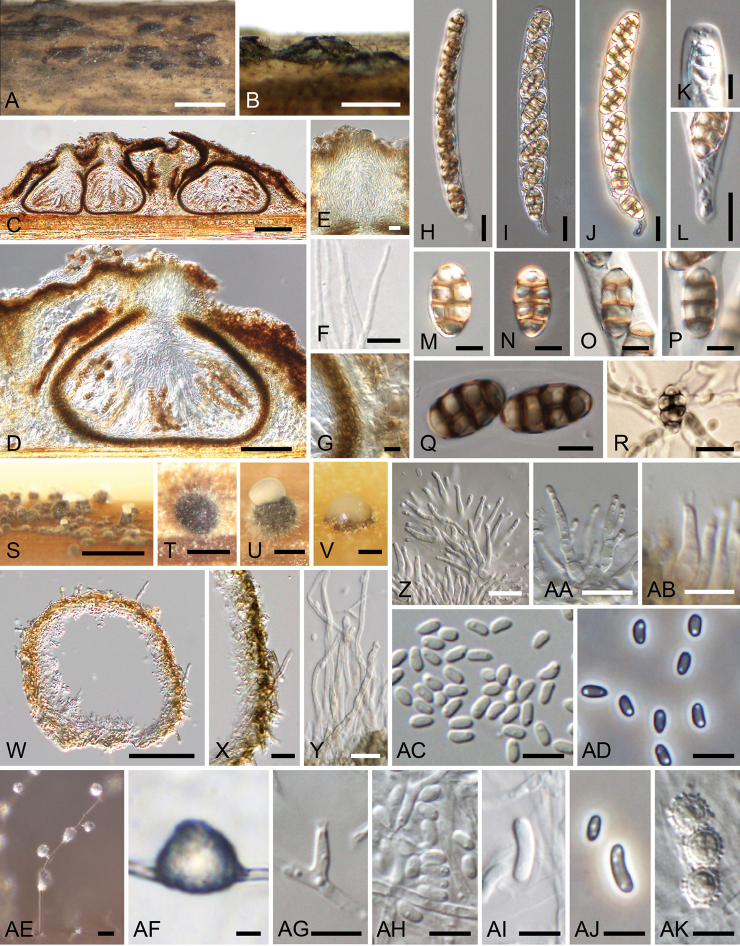
*Thyridiumpunctulatum* (**A–N, Q, R** KT 3905 = HHUF 30649 **O, P** YAM 21851 **S, T, W–AB** culture KT 1015 = JCM 13159 = MAFF 239669 **U, V, AC–AK** culture KT 3905 = MAFF 247510) **A–R** sexual morph **A, B** appearance of stromata on substrate **C, D** ascomata in longitudinal section **E** ostiolar neck of ascoma **F** paraphyses **G** ascomatal wall **H–J** asci **K** apex of ascus **L** stipe of ascus **M–Q** ascospores **R** germinating ascospore **S–AD** coelomycetous asexual morph **S–V** conidiomata in culture **W** conidioma in longitudinal section **X** conidiomatal wall **Y** setose hyphae of conidiomata **Z, AA** conidiophores **AB** phialides **AC, AD** conidia **AE–AK** hyphomycetous synasexual morph **AE** conidiophore **AF** slimy head **AG** phialide **AH–AJ** conidia **AK** chlamydospores. Scale bars: 1 mm (**A, S**); 500 µm (**B**); 100 µm (**C, W**); 50 µm (**D**); 10 µm (**E–J, L, R, X–AA, AE, AF**); 5 µm (**K, M–Q, AB–AD, AG–AK**); 200 µm (**T–V**).

##### Asexual morph (nature).

Not observed.

##### Asexual morph (culture).

Coelomycetous asexual morph: Conidiomata pycnidial, single to grouped, superficial, globose to subglobose, 100–250 µm high, 170–620 µm diam., composed of polygonal to prismatic, 3.5–7.5 × 2.5–4 µm cells, often becoming cup-shaped when mature, surrounded by setose hyphae. Setose hyphae erect, usually unbranched, septate, up to 225 µm long, 1.5–2.5 µm wide, pale brown. Conidiophores hyaline, thin-walled, simple or irregularly branched, with branches bearing a group of 2–5 phialides terminally. Phialides swollen at the base, tapering at the tip, 7–20 × 1–3 µm, hyaline. Conidia ellipsoidal to obovoid, with a slightly apiculate base, hyaline, smooth-walled, in slimy masses, 2–3.5 × 1–2 µm (av. 2.9 × 1.4 µm, n = 50). Hyphomycetous synasexual morph: Conidiophores micronematous, mononematous, hyaline, thin-walled, simple or irregularly branched, with branches bearing a group of 2–3 phialides terminally. Phialides swollen at the base, tapering at the tip, hyaline, 3–9 × 1–2 µm. Adelophialide absent. Conidia ellipsoidal to allantoid, hyaline, smooth-walled, in slimy heads, 2.5–8 × 1–3 µm (av. 4.3 × 1.6 µm, n = 87). Chlamydospores rarely present, solitary or chained, 4–5.5 µm diam., hyaline to pale brown.

##### Culture characteristics.

**Figure 6.**
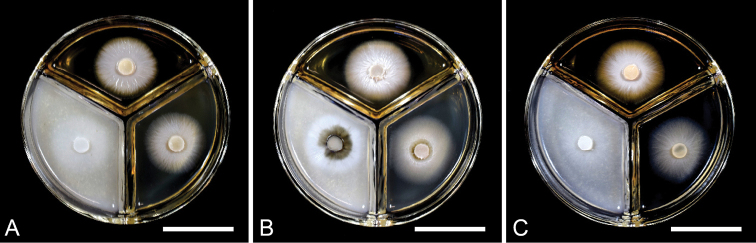
Colony characters of *Thyridium* species used in this study on MEA (bottom right), OA (bottom left) and PDA (upper) within 1 week at 25 °C in the dark **A***T.flavostromatum* (culture KT 3891 = MAFF 247509) **B***T.pluriloculosum* (culture KT 3803 = MAFF 247508) **C***T.punctulatum* (culture KT 3905 = MAFF 247510). Scale bars: 3 cm (**A–C**).

Colonies on MEA at 25 °C attained 31–32 mm diam. after a week in the dark, granulose, whitish. On OA attained 38–39 mm diam., granulose, whitish. On PDA attained 35–36 mm diam., whitish to buff (45) (Fig. 6C).

##### Other specimen examined.

Japan, Iwate, Morioka, Ueda, Campus of Iwate University, on dead culms of *Phyllostachyspubescens*, 17 February 2003, K. Tanaka and Y. Harada, KT 1015 (HHUF 29350), living culture JCM 13159 = MAFF 239669.

##### Notes.

This species has been described from Phyllostachysnigravar.henonis, as a species of *Pleospora* (Dothideomycetes; Hino 1961). Our phylogenetic analysis (Fig. 1) shows that this species is a member of the genus *Thyridium* (Sordariomycetes). The morphological features of this species are consistent with those of the genus *Thyridium*, including immersed to erumpent, single to grouped, perithecial ascomata with a cylindrical ostiolar neck, unitunicate asci and muriform, pigmented ascospores (Eriksson and Yue 1989). Therefore, we propose a new combination, *T.punctulatum*, for *Pleosporapunctulata*.

#### ﻿ Thyridium cornearis

Taxon classificationFungiThyridialesThyridiaceae

(Perdomo, Dania García, Gené, Cano & Guarro) R. Sugita & Kaz. Tanaka comb. nov.

735D2628-70C9-5F95-BD7E-A3E3C3D07FE8

841920

##### Basionym.

*Phialemoniopsiscornearis* Perdomo, Dania García, Gené, Cano & Guarro, Mycologia 105: 408 (2013).

#### ﻿ Thyridium curvatum

Taxon classificationFungiThyridialesThyridiaceae

(W. Gams & W.B. Cooke) R. Sugita & Kaz. Tanaka comb. nov.

08600168-16C1-5268-A21E-D60246B90A62

841921

Phialemoniopsis curvata (W. Gams & W.B. Cooke) Perdomo, Dania García, Gené, Cano & Guarro, Mycologia 105: 410 (2013).

##### Basionym.

*Phialemoniumcurvatum* W. Gams & W.B. Cooke, Mycologia 75: 980 (1983).

#### ﻿ Thyridium endophyticum

Taxon classificationFungiThyridialesThyridiaceae

(Lei Su & Y.C. Niu) R. Sugita & Kaz. Tanaka comb. nov.

6A1D7C2D-9A0B-5462-BE3B-43845F60A317

841922

##### Basionym.

*Phialemoniopsisendophytica* Lei Su & Y.C. Niu, Mycol. Progr. 15: 3 (2016).

#### ﻿ Thyridium hongkongense

Taxon classificationFungiThyridialesThyridiaceae

(Tsang, Chan, Ip, Ngan, Chen, Lau, Woo) R. Sugita & Kaz. Tanaka comb. nov.

4D4CC3F3-BDE6-5305-988F-3DA602F2E28E

841923

##### Basionym.

*Phialemoniopsishongkongensis* Tsang, Chan, Ip, Ngan, Chen, Lau, Woo, J. Clin. Microbiol. 52: 3284 (2014).

#### ﻿ Thyridium limonesiae

Taxon classificationFungiThyridialesThyridiaceae

(A. Riat, L.W. Hou & Crous) R. Sugita & Kaz. Tanaka comb. nov.

D61E332A-DA1F-55F0-B0F5-4F51020778AE

841927

##### Basionym.

*Phialemoniopsislimonesiae* A. Riat, L.W. Hou & Crous, Emerging Microbes & Infections 10: 403 (2021).

#### ﻿ Thyridium oculorum

Taxon classificationFungiThyridialesThyridiaceae

(Gené & Guarro) R. Sugita & Kaz. Tanaka comb. nov.

EAD7413C-C1B3-54EA-ACC8-BFF56F3ED0F0

841924

Phialemoniopsis ocularis (Gené & Guarro) Perdomo, Dania García, Gené, Cano & Guarro, Mycologia 105: 411 (2013).

##### Basionym.

*Sarcopodiumoculorum* Gené & Guarro, J. Clin. Microbiol. 40: 3074 (2002).

## ﻿Discussion

We show that the asexual genus *Phialemoniopsis* (established by Perdomo et al. 2013) is a synonym of the sexual genus *Thyridium* (established by Nitschke 1867). We found a new species of *Thyridium* (*T.flavostromatum*), transferred *Pleosporapunctulata* into *Thyridium*, and proposed seven new combinations in *Thyridium* for strains previously treated in *Phialemoniopsis*. We provided a revised generic circumscription of *Thyridium* based on both sexual and asexual characteristics and revealed the phylogenetic relationships of species within this genus.

The genus *Thyridium* has been defined mainly on the basis of sexual characters (Nitschke 1867; Eriksson and Yue 1989). Currently, 33 species are recorded in this genus (http://www.indexfungorum.org, 2021). Asexual morphs are unknown in most species of *Thyridium*, with the exceptions of *T.flavum* and *T.vestitum*, in which asexual morphs have been recorded based on sexual-asexual association on the same specimen (Petch 1917) and on the basis of culture study (Leuchtmann and Müller 1986, this study), respectively. In contrast, the genus *Phialemoniopsis* has been defined based only on asexual characters (Perdomo et al. 2013). Its ordinal affiliation within Sordariomycetes has not been resolved, but recent phylogenetic analyses of this class suggest that *Phialemoniopsis* is close to *Thyridium* (Hyde et al. 2021). In our phylogenetic analysis, all species previously described as *Phialemoniopsis* (marked with blue circle; Fig. 1) were clustered in a single clade, including the type species of *Thyridium* (*T.vestitum*), as well as two new strains proposed here (*T.flavostromatum* and *T.punctulatum*). Both genera have similar asexual morphs, which have conidiophores bearing small groups of phialides, hyaline phialidic conidiogenous cells, and ellipsoidal or allantoid, hyaline conidia in both coelomycetous and hyphomycetous states (Petch 1917; Leuchtmann and Müller 1986; Perdomo et al. 2013). Morphological and molecular phylogenetic evidence clearly shows that *Phialemoniopsis* is congeneric with *Thyridium*.

Synonymising *Phialemoniopsis* under *Thyridium* expanded information about the asexual morphs of *Thyridium*. In this genus, only *T.vestitum* has been demonstrated to have asexual morphs by culture studies (Leuchtmann and Müller 1986). It has both coelomycetous and hyphomycetous complex asexual morphs, which have phialidic conidiogenous cells with collarette and ellipsoidal to allantoid hyaline conidia (Leuchtmann and Müller 1986). Members of *Phialemoniopsis* also have coelomycetous and/or hyphomycetous conidial states (Perdomo et al. 2013; Tsang et al. 2014; Su et al. 2016; Martinez et al. 2021). The close relationship of *Phialemoniopsis* and *Thyridium* suggests that such complex asexual morphs may be common within *Thyridium* species.

In *Thyridium*, *T.endophyticum* and *T.curvatum* have been isolated from both plants and animals (Gam and McGinnis 1983; Halleen et al. 2007; Perdomo et al. 2013; Su et al. 2016; Ito et al. 2017). There are several examples of fungal species, including human pathogens, detected from various substrates. For example, *Phaeoacremoniumminimum* is a pathogen on grapevines, where it forms both sexual and asexual morphs (Crous et al. 1996; Pascoe et al. 2004), but it has also been reported as a causative agent of subcutaneous phaeohyphomycosis in humans as asexual morph (Choi et al. 2011). Other species of *Thyridium* may also have cryptic life cycles and can colonise each host substrate at different reproductive stages. An example of this prediction can be found in *T.pluriloculosum*. This species was originally found in human nails as an asexual fungus (Perdomo et al. 2013), and its sexual state was rediscovered on twigs of *Betulamaximowicziana* in our study.

Epitypification of the type species of *Thyridium* (*T.vestitum*) will be a necessary issue in the future. We used sequences from two non-type strains (CBS 113027, CBS 125582) of this species for phylogenetic analyses but they did not form a monophyletic clade (Fig. 1). Sequence differences between these two strains were found at 34 positions with four gaps in the LSU. These results indicate that the strains obtained from *Acerpseudoplatanus* (CBS 113027) and no host information (CBS 125582) in Austria are not conspecific. A fresh collection of *T.vestitum* on original host plant from the type locality (*Ribesrubrum*, Sweden; Fries 1823) and its phylogenetic analysis are required to fix generic circumscription of *Thyridium*.

Thyridiales established here may encompass other genera and families with morphologies distinct from the genus *Thyridium* (Thyridiaceae). Some species of “*Linocarpon*” and “*Neolinocarpon*” are nested within the Thyridiales (Fig. 1). *Linocarpon* and *Neolinocarpon* sensu stricto belong to Linocarpaceae (Chaetosphaeriales) and are morphologically distinct from *Thyridium* in having filiform, straight or curved, unicellular, hyaline, or pale-yellowish ascospores (Huhndorf and Miller 2011; Konta et al. 2017). The “*Linocarpon*” and “*Neolinocarpon*” species phylogenetically unrelated to *Linocarpon* and *Neolinocarpon* sensu stricto may be new lineages in Thyridiaceae or belong to its own new undescribed family. However, we cannot clarify the phylogenetic/taxonomic relatedness of these atypical *Linocarpon*-like species because none of them are ex-types and their morphological information are unavailable. Further molecular phylogenetic study of these fungi based on protein-coding sequences and finding additional specimens/isolates of “*Linocarpon*” and “*Neolinocarpon*” species related to *Thyridium* will be necessary to clarify their taxonomic affiliation and better understand the concept of Thyridiales.

## Supplementary Material

XML Treatment for Thyridiales

XML Treatment for Thyridiaceae

XML Treatment for Thyridium

XML Treatment for Thyridium flavostromatum

XML Treatment for Thyridium pluriloculosum

XML Treatment for Thyridium punctulatum

XML Treatment for Thyridium cornearis

XML Treatment for Thyridium curvatum

XML Treatment for Thyridium endophyticum

XML Treatment for Thyridium hongkongense

XML Treatment for Thyridium limonesiae

XML Treatment for Thyridium oculorum
